# The Chronic Time Pressure Inventory: further assessment of factorial structure and validity

**DOI:** 10.7717/peerj.17373

**Published:** 2024-05-01

**Authors:** Andrew Denovan, Neil Dagnall, Ken Drinkwater

**Affiliations:** 1 People and Performance, The Manchester Metropolitan University, Manchester, United Kingdom; 2 Psychology, The Manchester Metropolitan University, Manchester, United Kingdom

**Keywords:** Chronic time pressure, Chronic Time Pressure Inventory, Convergent validity, Exploratory structural equation modelling, Personality, Time perspective

## Abstract

**Background:**

Chronic time pressure represents a prevalent concern within modern society, and effective measurement is crucial for research advancement. The Chronic Time Pressure Inventory (CTPI) has thus far demonstrated adequate psychometric properties. However, only two studies have examined the measure and evidence of its validity is limited. Accordingly, the current investigation, *via* two independent studies, assessed the factorial composition and validity (convergent/discriminant) of the CTPI.

**Methods:**

Study 1 (*N* = 398) examined competing factorial models and validity in relation to the Big Five personality traits (Extraversion, Agreeableness, Conscientiousness, Neuroticism, Openness). Study 2 (*N* = 358) replicated the analysis of factor structure and assessed validity in comparison with five time perspectives (Past Negative, Present Fatalistic, Future, Past Positive, Present Hedonistic). Participants across both studies completed standardized self-report measures capturing the variables.

**Results:**

Comparison of confirmatory factor analysis (CFA) and exploratory structural equation modelling (ESEM) factor solutions indicated that an ESEM bifactor model provided the strongest data-model fit. This included a general chronic time pressure component alongside specific subfactors of Feeling Harried and Cognitive Awareness of Time Shortage. All scale items reflected the general factor; however, some items loaded weakly on the intended specific factor. The CTPI is thus a robust indicator of chronic time pressure but needs refinement as a measure of the specific factors. Convergent/discriminant validity analyses inferred that the CTPI captured chronic time pressure as a related, but distinct, construct to perceived stress, and evidenced a relationship with theoretically associated constructs (Big Five personality traits and time perspective). Overall, the CTPI is a sound measure of chronic time pressure and has the potential to further cohesive research efforts on the contribution of this construct to various life domains.

## Introduction

Within contemporary Western culture the subjective experience of time shortage is a major source of stress and anxiety (Gunthorpe & Lyons, 2004; Roxburgh, 2004; Southerton, 2020). This manifests typically as insufficient time to complete tasks, accompanied by feelings of being rushed and harried (Szollos, 2009). Noting the potentially harmful effects of time pressure on individuals and society, researchers have undertaken extensive multi-disciplinary research (Rudd, 2019). Whilst this produced useful theoretical insights, the breadth of approaches employed resulted in a diverse range of terminology and definitions. These created conceptual obfuscation, restricting the usefulness of generalizations across studies. To establish theoretical clarity, Szollos (2009) performed a review of previous academic work. This recommended the adoption of the term chronic time pressure (CTP). CTP was a significant conceptual advancement because the delineation synthesized preceding empirical work and provided a precise operationalization.

Szollos (2009, p.339) defined CTP as, “a temporary, overarching designation that would subsume all the terms related to time shortage as well as to being rushed.” This assumes the existence of a main CTP dimension alongside two components, time shortage (awareness of limited time) and sense of being rushed (feeling harried). Commensurate with these factors, CTP if acute or sustained is potentially psychologically and physically harmful. Time shortage refers to time management and cognizance of temporal restrictions and/or poverty. Hence, this dimension encompasses cognitive appraisal and objective assessment of time allocation in the absence of affect; whereas, being rushed is an experiential, emotional experience, which involves emotions/feelings of worry, frustration, and anxiety.

A further feature of the Szollos (2009) classification is that it frames time pressure within the context of stress-related theory (*i.e*., transactional theory, Lazarus & Folkman, 1984). Thus, perceived time pressure arises from the individual’s interaction with their environment. Explicitly, their attempts to complete tasks in self and externally generated time constrained windows. The allying of time pressure with stress emphasizes the ‘chronic’ effects that time pressure can have on individuals. In particular, it represents CTP as persistent, reoccurring, and predominantly undesirable.

Drawing on this conceptualization, Denovan & Dagnall (2019) developed the domain-general (*i.e*., context free) Chronic Time Pressure Inventory (CTPI). Item construction was guided by the work of Roxburgh (2004), whose Time Pressure Scale informed development of the items capturing notions of feeling rushed. The CTPI was necessary because preceding measures lacked coherence (agreed definition and terminology), and consequently there was no standard, extant psychometrically validated instrument. The use of myriad measurement tools was problematic as it limited the ability to draw comparisons between investigations. Additionally, previous scales sampled only narrow aspects of construct domain (*i.e*., used single or restricted numbers of items) (*e.g*., Ackerman & Gross, 2003; Kleiner, 2014). Despite this, self-report measures generally are a widely accepted and valid method for assessing perceived time pressure.

The CTPI was developed using apposite statistical procedures (*i.e*., parallel analysis, exploratory factor analysis, and confirmatory factor analysis with multigroup invariance tests). Consistent with Szollos (2009), analyses evidenced a bifactor structure comprising a general overarching time pressure factor alongside two distinct but overlapping subfactors (Feeling Harried and Cognitive Awareness of Time). This aligned with Szollos’s (2009) definition. The CTPI was internally consistent, possessing satisfactory omega reliability. Additionally, the CTPI demonstrated convergent validity *via* a strong positive association with perceived stress. This indicated that the CTPI captured additional variance beyond stress. This concurred with the delineation of CTP as temporally related stress (Szollos, 2009). Moreover, Denovan et al. (2023) discovered that the measure possessed adequate psychometric properties *via* Rasch analysis (*i.e*., reliability, lack of differential item functioning) alongside unidimensionality, supporting a dominant general component.

### The present study

To further establish the psychometric properties of the CTPI, this article tested (using two independent samples) the factorial composition of the scale and assessed the degree to which scores converged and diverged with theoretically related constructs. In Study 1, this was the Perceived Stress Scale (PSS, Cohen, Kamarck & Mermelstein, 1983) and the Big Five Inventory (BFI-44, John, Donahue & Kentle, 1991), and in Study 2, PSS and the Zimbardo Time Perspective Inventory (ZTPI, Zimbardo & Boyd, 1999). The PSS was selected to enable direct comparisons with the validation study. Consistent with Denovan & Dagnall (2019), it was predicted that PSS would strongly, positively correlate with overall CTPI and both sub-factors (*i.e*., Feeling Harried and Cognitive Awareness of Time).

The choice of the BFI-44 was informed by the observation that personality traits are differently related to stress. Although, there are variations across studies, previous research generally indicates that Neuroticism (*i.e*., negative emotionality) is positively related to stress, whereas Extraversion (*i.e*., energetic approach toward the social and material world), Agreeableness (*i.e*., prosocial and communal orientation), Conscientiousness (*i.e*., tendency to follow socially prescribed norms for impulse control, delay gratification, plan and follow norms and rules), and Openness (*i.e*., breadth, depth, originality, and complexity of mental and experiential life) are negatively associated (see Luo et al., 2022).

Noting this, it was hypothesized that the CTPI because of its strong relationship with perceived stress would demonstrate similar associations with personality traits. There were two caveats to this. Firstly, although CTP is related to stress it is a distinct construct and associations with BFI-44 dimensions may vary. Secondly, the effect sizes for associations between BFI-44 traits and stress across the literature are larger for Neuroticism, Extraversion, Agreeableness, and Conscientiousness than Openness (see Luo et al., 2022). This occurs because the relationship between stress and Openness is affected by factors such as stress conceptualization (*i.e*., exposure, psychological and physiological) as evidenced by mixed findings across studies (Luo et al., 2022).

The ZTPI (Zimbardo & Boyd, 1999) was used in Study 2 because it includes five time perspectives (Past Negative, Present Fatalistic, Future, Past Positive, and Present Hedonistic), which should demonstrate differential relationships with CTPI. It was expected that CTPI would correlate positively with undesirable perceptions of time (Past Negative, aversive view of past occurrences; and Present Fatalistic, hopeless attitudes towards the present), and negatively with optimistic/constructive temporal perspectives (Past Positive, sentimentality for the past; and Future (focus on future goals/rewards/ambitions)). Given temporal neutrality of Present Hedonistic (inclination to focus on pleasure with little regards for outcomes) no association was anticipated with CTPI.

The inclusion of the PSS within the present study allowed replication of the outcomes reported in the validation study. Additionally, Study 1 (BFI-44) and Study 2 (ZTPI) enabled the researchers to assess the convergent and discriminant validity of the CTPI.

## Study 1

### Materials and Methods

#### Sample

The sample comprised 398 participants (*M*age = 26.06 years, *SD* = 11.69, range 18–68), 323 females (81%, *M*age = 28.64 years, *SD* = 12.48, range 18–68) and 74 males (18.5%, *M*age = 25.47 years, *SD* = 11.46, range 18–67). One participant did not disclose their gender (age = 25). Of the sample, 97 (24%) participants were UK university students, 223 (56%) in full-time employment, 108 (28%) in part-time employment, and 62 (16%) unemployed.

#### Measures

**The Chronic Time Pressure Inventory (CTPI).** The CTPI (Denovan & Dagnall, 2019) is a 13-item self-report measure that assesses perceived chronic time pressure (*e.g*., ‘I feel pressured to fit everything in’). Items appear as statements and are presented alongside a Likert-type response format, which ranges from 1 (Strongly disagree) to 5 (Strongly agree). The CTPI consistent with Szollos (2009), comprises two factors: Feeling Harried and Cognitive Awareness of Time. The CTPI possesses satisfactory psychometric properties (Denovan & Dagnall, 2019). In this study, coefficient ω was good for the total scale (0.86) and for each subscale (Feeling Harried = 0.83, Cognitive Awareness of Time Shortage = 0.80).

**The 10-item Perceived Stress Scale (PSS-10).** The PSS-10 (Cohen, Kamarck & Mermelstein, 1983) includes 10 statements assessing an individual’s appraisal of stress within their life (*e.g*., ‘how often have you felt nervous and stressed?’). This focuses on the extent to which life is perceived as overloading, distressful and unmanageable, and uses the past month as the timeframe of focus. Participants respond on a 0 (Never) to 4 (Very often) scale. Subscales of PSS Distress (helplessness) and PSS Coping (efficacy) exist. The PSS-10 is a widely used and psychometrically validated measure (Denovan et al., 2019). Good coefficient ω existed for the total scale (0.91), and for PSS Distress (0.87) and PSS Coping (0.79) in the present investigation.

**The 44-item Big Five Inventory (BFI-44).** The BFI-44 (John, Donahue & Kentle, 1991) is a 44-item scale that assesses individuals on the Big Five components of personality. The measure includes a subscale for each of the five dimensions of Extraversion, Agreeableness, Conscientiousness, Neuroticism, and Openness. Statements focus on general characteristics (*e.g*., ‘I see myself as someone who is talkative’) and include a five-point Likert type response format (from ‘1 = Disagree strongly’ to ‘5 = Agree strongly’). Good psychometric properties exist for its subscales (*e.g*., Benet-Martínez & John, 1998). In this study, good coefficient ω existed for Extraversion (0.87), Agreeableness (0.76), Conscientiousness (0.82), Neuroticism (0.86), and Openness (0.80).

#### Procedure and ethics

Recruitment used Qualtrics, an online data collection platform. Participants were sent the information sheet *via* a web-link. This detailed the study background and requested informed consent. Participants who provided written consent continued to the instructions, which emphasized the importance of honest and open responses. Measures included a demographics component (*i.e*., age, preferred gender) alongside the scales. Participants were debriefed following involvement.

Procedural techniques were included to minimize participant bias, such as emphasizing there were no right or wrong answers (for social desirability) and scale rotation (to counter order effects) (see Dagnall, Denovan & Drinkwater, 2022; Dagnall et al., 2022). Ethical approval was granted by the Faculty of Health, Psychology and Social Care Ethics Committee at Manchester Metropolitan University (Project ID: 2058).

#### Analysis

Analyses were performed with Mplus (version 7.3, Muthén & Muthén, 2014) and SPSS 28 (SPSS, Inc., Chicago, IL, USA). Following data screening, a range of factorial models were compared. These included confirmatory factor analysis (CFA) one-factor (as a null test), two-factor correlated (with Feeling Harried, FH and Cognitive Awareness of Time Shortage, CA components), and bifactor (comprising a general factor alongside FH and CA components) solutions. A two-factor and bifactor model were additionally tested using exploratory structural equation modelling (ESEM). The use of ESEM was an important development for assessing the CTPI’s psychometric properties. Explicitly, the initial validation study (Denovan & Dagnall, 2019) used CFA. A problem with CFA is that fixing cross-loadings to zero can inflate associations among latent factors (Espinoza et al., 2018). ESEM allows items to cross-load on factors, and due to its flexibility and less restrictive approach often produces better data fit (Morin, Arens & Marsh, 2016).

Assessment of data-model fit used multiple goodness-of-fit statistics: comparative fit index (CFI), root-mean-square error of approximation (RMSEA), standardized root-mean-square residual (SRMR) and Akaike Information Criteria (AIC). Guidelines by Hu & Bentler (1999) recommend CFI > 0.90 for satisfactory fit, and >0.95 for good fit. RMSEA and SRMR < 0.08 suggests satisfactory fit, and <0.05 denotes good fit (Browne & Cudeck, 1993). For model comparison, Chen (2007) advises that CFI increases between 0.005–0.010 and RMSEA decreases between 0.010–0.015 reflect a meaningful improvement in data fit. Lower AIC values additionally indicate better fit. Factor loadings >0.30 are considered acceptable (Gliner, Morgan & Leech, 2016). Target loading was implemented for ESEM, whereby all items are permitted to load on all components, with cross-loadings for items on non-corresponding components estimated to be adjacent as possible to zero. All analyses used robust Maximum Likelihood estimation, which calculates fit indices and standard errors that are robust to normality violations (Finney & DiStefano, 2013).

Subsequent tests of convergent/discriminant validity examined associations between the CTPI, PSS, and BFI-44 factors (Extraversion, Agreeableness, Conscientiousness, Neuroticism, Openness). Moreover, partial correlations (controlling for PSS) were assessed to determine the unique contribution of CTPI to BFI-44 factors in the absence of PSS. Correlation strength interpretation used the guidelines of Gignac & Szodorai (2016).

### Study 1 results

#### Factor analysis

Data screening revealed satisfactory skewness and kurtosis (*i.e*., within the tolerance of −2 to +2; Byrne, 2010). The one-factor model demonstrated unsatisfactory fit on CFI, χ^2^ (65) = 263.34, *p* < 0.001, CFI = 0.84, RMSEA = 0.08 (0.07, 0.09), SRMR = 0.06. The two-factor correlated model, comprising Feeling Harried and Cognitive Awareness of Time Shortage factors, also reported acceptable RMSEA and SRMR and a 0.06 CFI improvement, χ^2^ (64) = 190.31, *p* < 0.001, CFI = 0.90, RMSEA = 0.07 (0.05, 0.08), SRMR = 0.05. The bifactor model, χ^2^ (52) = 120.76, *p* < 0.001, CFI = 0.94, RMSEA = 0.05 (0.04, 0.07), SRMR = 0.03, demonstrated superior fit across indices and on AIC *vs*. the two-factor correlated model (13,574.70 *vs*. 13,646.53). However, CFI was <0.95. The two-factor ESEM solution evidenced improved fit in comparison, χ^2^ (53) = 113.89, *p* < 0.001, CFI = 0.95, RMSEA = 0.05 (0.04, 0.06), SRMR = 0.03. Specifically, a 0.006 increase in CFI, 0.004 decrease in RMSEA alongside lower chi-square and AIC (13,572.80 *vs*. 13,574.70).

The bifactor ESEM model, χ^2^ (42) = 103.35, *p* < 0.001, CFI = 0.95, RMSEA = 0.06 (0.04, 0.07), SRMR = 0.02, evidenced a 0.007 decrease in SRMR, lower chi-square and AIC (13,556.49 *vs*. 13,572.80) compared with the ESEM solution. Though, a 0.007 increase in RMSEA existed, the bifactor ESEM model demonstrated better data fit overall. In instances of similarities in fit (*i.e*., *versus* the two-factor ESEM model), Morin, Arens & Marsh (2016) emphasize the need to closely scrutinize theoretical conformity and parameter estimates when identifying the superior model. This is especially important when fit indices are similar.

Comparison of factor loadings (Table 1) for the ESEM and bifactor ESEM models revealed considerably smaller cross-loadings for the latter solution. These supported the existence of an unmodeled general dimension evidenced by higher cross-loadings in the ESEM solution. Specifically, there existed five statistically significant cross-loadings in the ESEM model, whereas only one significant cross-loading occurred in the bifactor ESEM model. In addition to the fit indices, this indicated that the bifactor ESEM was superior.

**Table 1:** Standardized bifactor ESEM factor loadings for Study 1.

				Bifactor	
Scale	Sub-scale	Item	General factor	FH	CA
CTP	FH	4. The days fly by without me ever getting everything done	0.43	0.28	*−0.03*
		8. I worry about how well I use my time	0.40	0.51	*0.06*
		10. I think I won’t finish work that I set out to do	0.61	0.48	*0.02*
		11. I feel disappointed with how I spend my time	0.62	0.59	*−0.03*
		12. I always run out of time	0.57	0.40	*0.01*
	CA	1. There aren’t enough hours in the day	0.51	*−0.08*	0.32
		2. I have enough time to do the things that I want to do	0.40	*−0.05*	0.33
		3. I feel pressured to fit everything in	0.32	*0.01*	0.18
		5. I am often in a hurry	0.51	*−0.18*	0.19
		6. I feel in control of how I spend my time	0.45	*0.29*	0.37
		7. I should have more free time to do the things I enjoy	0.42	*−0.17*	0.36
		9. I have enough time to properly prepare for things	0.64	*0.09*	0.36
		13. I feel rushed to do the things that I have to do	0.78	*0.03*	−0.11
*Mean* λ			0.51	0.45	0.25

**Note:**

Italicized items represent non-target loadings. CTP, chronic time pressure, FH, feeling harried, CA, cognitive awareness of time shortage.

The loadings for the bifactor ESEM general factor designated a well-defined dimension (λ = 0.32 to 0.78, *M*_λ_ = 0.51). The Feeling Harried factor was reasonably well-defined (λ = 0.28 to 0.59, *M*_λ_ = 0.45), yet Item 4 loaded below 0.32 (0.28). Cognitive Awareness of Time Shortage was comparatively poorly defined (λ = −0.11 to 0.37, *M*_λ_ = 0.25), with three of the eight target items displaying significant loadings and no Feeling Harried items evidencing significant cross-loading. The lack of significant loadings among target and non-target items specified that the associated variance was mainly utilized in defining the general dimension.

Consideration of bifactor-specific criteria (Explained Common Variance, ECV, and hierarchical omega, ω_h_) determined the strength of the general factor. ECV > 0.60 and ω_h_ > 0.70 suggest unidimensionality (Reise, Bonifay & Haviland, 2013). The ECV was 0.67, and the ω_h_ coefficient of 0.73 for the general factor alongside ω_hs_ coefficients of 0.35 and 0.16 for Feeling Harried and Cognitive Awareness of Time Shortage additionally supported a strong, general component.

#### Convergent/discriminant validity

The CTPI demonstrated moderate to large negative associations with Extraversion, Agreeableness, and Conscientiousness using the guidelines of Gignac & Szodorai (2016) (Table 2). In addition, CTPI correlated strongly and positively with Neuroticism and Perceived Stress. A non-significant association existed with Openness. When PSS scores were partialled out, relationships with Extraversion, Agreeableness, Conscientiousness, and Neuroticism, decreased slightly in strength and maintained significance. Explicitly, CTPI with Extraversion (*r* = −0.17, *p* < 0.001), Agreeableness (*r* = −0.19, *p* < 0.001), Conscientiousness (*r* = −0.33, *p* < 0.001), Neuroticism (*r* = 0.31, *p* < 0.001).

**Table 2:** Descriptive statistics and correlations among Study 1 variables.

Variable	*M*	*SD*	Skew.	Kurt.	1	2	3	4	5	6	7	8	9
1. CTP	42.15	6.27	−0.04	−0.05									
2. FH	16.29	3.99	−0.11	−0.32	0.88**								
3. CA	26.42	5.10	−0.05	−0.33	0.77**	0.56**							
4. PSS	31.29	3.53	−0.11	−0.41	0.51**	0.44**	0.40**						
5. Extraversion	24.93	6.12	−0.01	−0.50	−0.20**	−0.24**	−0.08	−0.11**					
6. Agreeableness	33.53	5.05	−0.56	0.20	−0.26**	−0.28**	−0.17**	−0.19**	0.19**				
7. Conscientiousness	30.43	5.60	−0.05	−0.18	−0.36**	−0.47**	−0.17**	−0.15**	0.22**	0.27**			
8. Neuroticism	25.66	6.27	−0.15	−0.33	0.50**	0.48**	0.38**	0.54**	−0.34**	−0.32**	−0.35**		
9. Openness	34.15	5.89	−0.18	0.24	−0.05	−0.04	−0.04	−0.02	0.25**	0.06	0.12*	−0.20**	

**Notes:**

Raw average scores are displayed across variables; CTP, chronic time pressure; FH, feeling harried; CA, cognitive awareness of time shortage; PSS, perceived stress.

* *p* < 0.05.

** *p* < 0.001.

CTPI subscales evidenced slightly different relationships with personality traits. Feeling Harried reported a similar pattern to total CTPI. Specifically, negative moderate to large associations with Extraversion, Agreeableness, Conscientiousness (not Openness), and a large positive correlation with Neuroticism and Perceived Stress. Partialling out PSS scores resulted in slightly reduced but still significant relationships: Feeling Harried with Extraversion (*r* = −0.22, *p* < 0.001), Agreeableness (*r* = −0.22, *p* < 0.001), Conscientiousness (*r* = −0.46, *p* < 0.001), and Neuroticism (*r* = 0.32, *p* < 0.001).

Cognitive Awareness of Time Shortage demonstrated small negative correlations with Agreeableness and Conscientiousness (not Extraversion nor Openness). Large positive associations existed with Neuroticism and Perceived Stress. Controlling for PSS reduced the relationships with Agreeableness (*r* = −0.11, *p* = 0.03), Conscientiousness (*r* = −0.12, *p* = 0.01), and Neuroticism (*r* = −0.22, *p* < 0.001). However, these remained significant.

#### Conclusion

Findings supported a dominant general CTP dimension, with some non-redundant support for the Feeling Harried factor. Correlations indicated a similar pattern of relationships between CTPI and BFI-44 factors as the PSS-10. Moreover, controlling for PSS scores produced slightly reduced but still significant relationships.

## Study 2

### Materials and Methods

#### Sample

The Study 2 sample comprised 358 participants (*M*age = 20.08 years, *SD* = 3.72, range 18–44). The sample was predominantly female (280, 78%, *M*age = 19.38 years, *SD* = 2.33, range 18–37). In total, 76 males (21%, *M*age = 22.01 years, *SD* = 5.04, range 18–37) participated, and two opted to not disclose gender (1%, *M*age = 43.50 years, *SD* = 0.71, range 43–44). Of the sample, 64 (17%) were in full-time employment, and 294 were UK university students, of which 122 (34%) were in part-time employment, and 172 (49%) were not employed.

#### Measures

As with Study 1, Study 2 utilized the CTPI and the PSS-10. Good ω existed for the CTPI (0.87) alongside the subscales Feeling Harried (0.79) and Cognitive Awareness of Time Shortage (0.81). Moreover, the PSS-10 evidenced strong ω reliability (0.90), as did PSS Distress (0.89) and PSS Coping (0.77).

**The Zimbardo Time Perspective Inventory (ZTPI).** The ZTPI (Zimbardo & Boyd, 1999) assesses cognitive, behavioral, and affective components of temporal psychology. The measure includes five time perspectives (see Introduction), which are presented as statements: Past Negative (*e.g*., ‘I often think of what I should have done differently in my life’), Present Hedonistic (*e.g*., ‘I believe that getting together with one’s friends to party is one of life’s important pleasures’), Future (*e.g*., ‘When I want to achieve something, I set goals and consider specific means for reaching those goals’), Past Positive (*e.g*., ‘Familiar childhood sights, sounds, smells often bring back a flood of wonderful memories’), and Present Fatalistic (*e.g*., ‘Fate determines much in my life’). The ZTPI comprises 56 items and participants respond *via* a five-point response format (1 = ‘Very unlike me’ to 5 = ‘Very like me’). The ZTPI possesses established, well evidenced psychometric properties (Zimbardo & Boyd, 1999). In this study, satisfactory to good omega occurred for Past Negative (0.87), Present Hedonistic (0.83), Future (0.76), Past Positive (0.81), and Present Fatalistic (0.71).

#### Procedure

The approach to recruitment and data collection/study procedure was similar to Study 1 and ethical approval was obtained from the same governing board.

#### Analysis

The same analytic approach was utilized as for Study 1; however, convergent validity analyses examined the CTPI and PSS in relation to ZTPI factors (Past Negative, Present Hedonistic, Future, Past Positive, Present Fatalistic).

### Study 2 results

#### Factor analysis

Data screening revealed acceptable skewness and kurtosis among study variables. The one-factor CTP solution reported unsatisfactory fit across all indices but SRMR, χ^2^ (65) = 297.32, *p* < 0.001, CFI = 0.83, RMSEA = 0.10 (0.08, 0.11), SRMR = 0.06. Unacceptable CFI existed for the two-factor model, χ^2^ (64) = 235.97, *p* < 0.001, CFI = 0.87, RMSEA = 0.08 (0.07, 0.09), SRMR = 0.05. The two-factor bifactor model, χ^2^ (52) = 169.81, *p* < 0.001, CFI = 0.91, RMSEA = 0.08 (0.06, 0.09), SRMR = 0.04, exhibited superior fit and reduced AIC (12,092.55 *vs*. 12,159.90). CFI remained below 0.95, as with Study 1. The two-factor ESEM solution also demonstrated CFI < 0.95, χ^2^ (53) = 174.19, *p* < 0.001, CFI = 0.91, RMSEA = 0.08 (0.06, 0.09), SRMR = 0.04. In contrast, the bifactor ESEM model evidenced good fit, χ^2^ (42) = 107.66, *p* < 0.001, CFI = 0.95, RMSEA = 0.06 (0.05, 0.08), SRMR = 0.03, alongside lower AIC than the two-factor ESEM and bifactor (12,046.81 compared with 12,103.12 and 12,092.55). Therefore, this indicated superior data fit.

Scrutiny of parameter estimates (Table 3) revealed a well-defined general CTP factor (λ = 0.34 to 0.89, *M*_λ_ = 0.53). Feeling Harried was poorly defined due mainly to Items 11 and 13 (loadings of 0.14 and −0.04), λ = −0.04 to 0.65, *M*_λ_ = 0.28. Cognitive Awareness of Time Shortage fared better (λ = 0.20 to 0.50, *M*_λ_ = 0.37). ECV of the general factor was 0.68, with a ω_h_ of 0.72. The ω_hs_ for the bifactors was lower (Feeling Harried = 0.20, Cognitive Awareness of Time Shortage = 0.25).

**Table 3:** Standardized bifactor ESEM factor loadings for Study 2.

				Bifactor	
Scale	Sub-scale	Item	General factor	FH	CA
CTP	FH	4. The days fly by without me ever getting everything done	0.41	0.26	*0.16*
		8. I worry about how well I use my time	0.34	0.65	*0.06*
		10. I think I won’t finish work that I set out to do	0.44	0.14	*0.04*
		11. I feel disappointed with how I spend my time	0.45	0.41	*−0.20*
		12. I always run out of time	0.49	−0.04	*−0.02*
	CA	1. There aren’t enough hours in the day	0.70	*−0.11*	0.37
		2. I have enough time to do the things that I want to do	0.39	*−0.03*	0.37
		3. I feel pressured to fit everything in	0.43	*0.22*	0.46
		5. I am often in a hurry	0.49	*−0.03*	0.40
		6. I feel in control of how I spend my time	0.71	*−0.08*	0.20
		7. I should have more free time to do the things I enjoy	0.56	*−0.18*	0.50
		9. I have enough time to properly prepare for things	0.89	*−0.01*	0.26
		13. I feel rushed to do the things that I have to do	0.56	*0.18*	0.40
*Mean* λ			0.53	0.28	0.37

**Note:**

Italicized items represent non-target loadings. CTP, chronic time pressure; FH, feeling harried;CA, cognitive awareness of time shortage.

#### Convergent/discriminant validity

The CTPI exhibited large positive correlations with Past Negative, Present Fatalistic, and PSS (Table 4). Small negative associations existed with Future and Past Positive (no significant link existed with Present Hedonistic). Controlling for PSS weakened the relationships concerning CTPI with Past Negative (*r* = 0.10, *p* = 0.058), Present Fatalistic (*r* = 0.13, *p* = 0.01), Future (*r* = −0.11, *p* = 0.03), and Past Positive (*r* = −0.11, *p* = 0.02). Specifically, the association with Past Negative became non-significant, whereas the other associations remained significant.

**Table 4:** Descriptive statistics and correlations among Study 2 variables.

Variable	*M*	*SD*	Skew.	Kurt.	1	2	3	4	5	6	7	8	9
1. CTP	42.72	8.29	−0.04	−0.05									
2. FH	16.93	3.90	−0.11	−0.32	0.86**								
3. CA	25.53	5.35	−0.05	−0.33	0.91**	0.61**							
4. PSS	31.14	7.46	−0.11	−0.41	0.65**	0.60**	0.56**						
5. Past negative	32.36	7.49	−0.01	−0.50	0.44**	0.46**	0.34**	0.58**					
6. Present hedonistic	51.82	7.65	−0.56	0.20	0.08	0.10*	0.08	0.01	0.23**				
7. Future	44.98	6.57	−0.05	−0.18	−0.14*	−0.23**	−0.06	−0.09	−0.14**	−0.43**			
8. Past positive	31.34	5.84	−0.15	−0.33	−0.15*	−0.14*	−0.11*	−0.37**	−0.43**	0.11*	0.07		
9. Present fatalistic	24.37	5.08	−0.18	0.24	0.31**	0.35**	0.24**	0.33**	0.48**	0.43**	−0.43*	−0.16*	

**Notes:**

Raw average scores are displayed across variables; CTP, chronic time pressure; FH, feeling harried; CA, cognitive awareness of time shortage; PSS, perceived stress.

* *p* < 0.05.

** *p* < 0.001.

As with Study 1, CTPI subfactors demonstrated slightly different associations. Feeling Harried was strongly positively associated with Past Negative, Present Fatalistic, and PSS, and small to moderate negative correlations with Future and Past Positive. Partitioning out PSS resulted in slightly weaker relationships that retained significance regarding Feeling Harried with Past Negative (*r* = 0.18, *p* < 0.001), Present Fatalistic (*r* = 0.20, *p* < 0.001), Future (*r* = −0.22, *p* < 0.001), and Past Positive (*r* = −0.11, *p* = 0.04).

Cognitive Awareness of Time Shortage evidenced moderate to large positive relationships with Past Negative, Present Fatalistic, and PSS. A weak negative association occurred relative to Past Positive, whereas non-significant associations existed with Future and Present Hedonistic. Controlling for PSS weakened the relationships with ZTPI considerably; only a significant correlation remained with Past Positive (*r* = −0.11, *p* = 0.02).

#### Conclusion

As with Study 1, a dominant general CTP dimension existed. Some non-redundant support occurred for the Cognitive Awareness of Time Shortage factor. Correlations indicated a similar pattern of relationships between CTPI and ZTPI factors as the PSS-10. Controlling for PSS scores resulted in slightly reduced but still significant relationships for total CTPI and Feeling Harried; however, a significant association existed with Past Positive only for Cognitive Awareness of Time Shortage.

## Overall discussion

Findings across two independent studies supported a dominant general CTP factor, with non-redundant support for two subfactors (Feeling Harried and Cognitive Awareness of Time Shortage). The general dimension captured negative aspects related to the subjective experience of time shortage. Support for a general factor aligned with the findings of Denovan & Dagnall (2019), and Denovan et al. (2023), and indicated the need to administer the full scale when collecting data, as opposed to utilizing the subfactors. In contrast with the original analyses (Denovan & Dagnall, 2019), this project employed ESEM in addition to CFA. The bifactor ESEM model outperformed the bifactor CFA model (on data-model fit), owing to its flexibility. Hence, the theoretical framework of CTP (from Szollos, 2009) was supported in the bifactor ESEM due to evidence for an overarching construct, on which the items loaded highly in addition to loading on their specific factors.

Explicitly, Feeling Harried items of 8 (‘I worry about how well I use my time’) and 11 (‘I feel disappointed with how I spend my time’) exhibited satisfactory loadings on the Feeling Harried factor in both studies, but the remaining items differed (items 10 and 12) or loaded poorly (item 4). A potential reason for this divergence is that items 8 and 11 possess prominent affective connotations (*i.e*., feelings of worry and disappointment), which are different in tone than the remaining items on the CTPI scale. It is likely that these items particularly capture core features of Szollos’s delimitation of feeling rushed. Cognitive Awareness of Time Shortage items loaded more prominently on the Cognitive Awareness of Time Shortage component in Study 2, but these were typically weaker than their respective loadings on the general CTP factor.

Thus, support existed for some unique variance of the subfactors and both factors possess considerable theoretical divergence (Denovan & Dagnall, 2019). Accordingly, researchers should administer the full measure rather than separate subscales and use subfactor scores in combination with the overall measure total (McElroy et al., 2018). Further development/refinement of the CTPI is necessary to remedy issues relating to items loading poorly on their intended factors. Currently, it is possible that scale scores are contaminated by overall chronic time pressure, which can result in under- or over-estimation of the specific factors’ associations with other constructs. It is likely that the bifactor ESEM was the best-fitting solution because it: (1) specified a general component to account for shared variance from aspects related to overall CTP; (2) contained specific components reflecting meaningful distinctions as described by Szollos (2009); and (3) permitted minor cross-loadings to explain any overlap in item content (Asparouhov & Muthén, 2009).

Convergent/discriminant validity analyses revealed strong, positive associations between the CTPI and PSS. This showed that the CTPI captured elements of general perceived stress. Moreover, the CTPI and PSS were similarly related to BFI-44 personality traits and ZTPI time perspectives. Explicitly, weak relationships with Openness, perhaps owing to the role of other facets including stress conceptualization (Luo et al., 2022), a strong negative association with Agreeableness and Conscientiousness, and a strong positive relationship with Neuroticism. Cognitive Awareness of Time Shortage (CA) did not correlate significantly with Extraversion. This may reflect the absence of items indexing assertiveness, drive, and energy. The relationship with Neuroticism likely reflected the fact that CTP indexes negative affect and cognitive concerns relating to situational demand (*i.e*., time). These elements also contribute to the strong relationship between PSS and Neuroticism (McCrae, 1990; Tananuvat et al., 2022).

As predicted, CTPI correlated differently with time perspectives (*i.e*., positively with Past Negative and Present Fatalistic, negatively with Past Positive, and weakly with Present Hedonistic). Future was significantly negatively correlated with CTPI and Feeling Harried (FH), however, there was no significant relationship with CA. The weaker relationship for CTPI specified that the relationship with Future was explained primarily by FH. This is possibly explained by the fact that the Future scale references positive outlook (Brothers et al., 2016). In contrast, FH indexes anxiety and frustration arising from the experience of the rapid passage of time.

Analyses of partial correlations (controlling for PSS) supported the notion that CTPI was uniquely related to BFI-44 and ZTPI dimensions. At the subscale level, partialling out PSS attenuated relationships between CA and ZTPI factors (Past Negative and Present Fatalistic). This reflected the nature of CA, which denotes objective appraisal of time shortage. This affect-free evaluation differs from FH, which involves perception of the negative, affective consequences of time pressure. These elements explain why in contrast to CA, FH correlated more strongly with Past Negative and Present Fatalistic factors, which respectively embody a focus on negative experiences in the past and the present (Simon et al., 2022). Overall, partial correlation revealed the CTPI explained unique variance in theoretically related constructs above and beyond the PSS.

### Limitations

The sample in Study 2 was comprised largely of university students (mean age 20.08 years), potentially limiting the generalizability of results to middle-aged and older adults in the general population. This is an important consideration because perception of time pressure changes across the life cycle (Fraisse, 1964). Moreover, ethnicity data was not collated. Cultural expectations are value-based factors that impact the experience of demand (a likely critical factor in the individual judgement of time pressure) (Tweed, White & Lehman, 2004). In addition, although data was collected from people in a range of occupations, working status/type was not a focus of the study. Time pressure can alter in association with occupation (Toppinen-Tanner, Kalimo & Mutanen, 2002), and it would be an important development for the CTPI to assess its performance more thoroughly in relation to different occupational strata/type. Lastly, both samples comprised a greater number of female than male participants. Although this potentially reflects a bias when considering the underpinning relationships among study constructs, it is important to note that a lower male response rate is not unique to research of this nature (Denovan et al., 2023). Indeed, research studies using traditional means of recruitment (*i.e*., word of mouth, snowball) often indicate comparable gender differences (*e.g*., Denovan et al., 2018; Lange et al., 2019).

## Conclusions

This article found that the CTPI is best represented by a bifactor ESEM model that includes a general overarching domain alongside factors of FH and CA. This designation is consistent with Szollos’s (2009) theory. Moreover, analysis confirmed that the CTPI is an appropriate tool for assessing perceptions of time pressure. However, until refinement of underperforming specific factor items takes place, it is recommended that the CTPI is administered in its entirety and that researchers use total scores. Once the factors are more fully developed, preliminary evidence exists to suggest that CTPI subscales will measure conceptually distinct but related elements of the perception of chronic time pressure. This is important since objective (CA) *vs*. affective (FH) components of time pressure are likely to interact with well-being in different ways. In this context, because the present study was cross-sectional, ensuing academic work should conduct longitudinal studies. These would establish the temporal stability of chronic time pressure and identify mediating and moderating variables. For instance, the effects of chronic time pressure on life satisfaction over time may change because of the presence (*e.g*., increases in life stress) and/or absence (*e.g*., social support) of particular factors. Moreover, this investigation demonstrated that the CTPI captured CTP appropriately as a related, but distinct, construct to perceived stress, and evidenced a relationship with theoretically associated constructs. It is therefore necessary for future research to implement the CTPI to capture chronic time pressure given it is a significant feature of everyday life (Southerton, 2020).

Moreover, although this research evidenced associations between CTP and theoretically meaningful constructs, an important future step would be to assess relationships with additional significant factors to progress the literature on the CTPI. For example, Roxburgh (2002, 2004, 2006, 2012) consistently identified the importance of social and demographic factors (*e.g*., income, gender) as moderators in the time pressure-mental health relationship. Comparably locating the CTPI within this would crucially add nomological validity. Relatedly, other features of personality and behavior are frequently identified as critical to the experience (or otherwise) of time pressure/stress, including perfectionism and procrastination (*e.g*., Shih, 2017). A necessary future step in research would be to examine how these interact with the CTPI.

## Additional Information and Declarations

### Data Availability

The following information was supplied regarding data availability: The datasets generated during and/or analysed are available at Figshare: Denovan, Andrew (2023). CTPI Data. figshare. Dataset. https://doi.org/10.6084/m9.figshare.22717993.v1.
